# Involvement of Ethylene in Adventitious Root Formation of Red-Stalked Rhubarb In Vitro

**DOI:** 10.3390/ijms26199429

**Published:** 2025-09-26

**Authors:** Agnieszka Wojtania, Piotr Waligórski, Monika Markiewicz

**Affiliations:** 1 Department of Applied Biology, The National Institute of Horticultural Research, Konstytucji 3 Maja 1/3 Str., 96-100 Skierniewice, Poland; monika.markiewicz@inhort.pl; 2 Department of Biotechnology, The Franciszek Górski Institute of Plant Physiology, Polish Academy of Sciences, 30-239 Kraków, Poland; pewalig7@gmail.com

**Keywords:** ABA, auxin, cytokinin content, gene expression, hormonal balance, *Rheum*

## Abstract

Irregular rooting in vitro is a major problem in the micropropagation of culinary rhubarb (*Rheum rhaponticum*), a vegetable crop rich in bioactive compounds. To date, little is known about the factors and mechanisms underlying adventitious root (AR) formation in rhubarb under in vitro conditions. Here, we studied the effects of indole-3-butyric acid (IBA) and its interaction with ethylene (ET) on AR development in rhubarb ‘Raspberry’ selection. To evaluate the ET-effect, we applied a precursor of ET biosynthesis—1 aminocyclopropane-1-carboxylic acid (ACC); an inhibitor of ET synthesis—aminoethoxyvinylglycine (AVG); and an inhibitor of ET action—silver nitrate (AgNO_3_). The best results (96.9% rooting frequency, 12.7 roots/shoot) were obtained after adding ACC to the IBA-containing medium. The positive effect of ET was linked to decreased levels of cytokinin and auxins in the rhubarb shoot bases at the initiation and expression stages of rooting. Moreover, the enhanced expression levels of genes involved in auxin signalling and homeostasis (*IAA17*, *GH3.1*) and ABA catabolism (*CYP707A1*) were observed. The blocking of ethylene synthesis significantly increased JA production, and the rooting frequency decreased to 29.8%. The presence of AgNO_3_ in the auxin medium resulted in a significant reduction in root number, which was consistent with the enhanced levels of ABA and the expression of genes related to ABA biosynthesis and signalling (*PP2C49* and *CBF4*), as well as ET synthesis (*ACO5*).

## 1. Introduction

Garden rhubarb (*Rheum rhaponticum,* synonyms: culinary rhubarb, edible rhubarb, vegetable-type rhubarb) is a vegetable crop primarily cultivated for its long, thickened leafy petioles (stalks), which are rich in phenolic compounds, mainly anthocyanins and flavonols, as well as numerous organic acids [1,2]. They are consumed in various forms, including fresh, frozen, freeze-dried, dehydrated, and processed and derived products, such as beverages, jams, juices, and wine. Rhubarb stalks are a valued ingredient in functional foods and dietary supplements [1,3,4]. Particular attention is now paid to red-stalked cultivars (both skin and flesh), which are richest in health-promoting compounds and more attractive to the processing industry than green-stalked rhubarb [5,6]. It has been reported that the levels of bioactive metabolites vary significantly among rhubarb genotypes [7]. Among the rhubarbs cultivated in Poland, the Raspberry type (Polish name Malinowy) is the richest in bioactive compounds, including cyanidin-3-O-rutinoside and rhaponticin [8]. Since rhubarb cultivars are highly heterozygous, maintaining desirable traits is only possible through asexual propagation. In vitro cultures are crucial for the rapid multiplication of valuable selections and for producing virus-free planting materials [9,10]. Unfortunately, most Raspberry rhubarb genotypes were characterized by irregular rooting in vitro [8]. The lack of rooting or deficiencies in root architecture reduce the survival rate and hinder the mass production of planting materials for selected genotypes.

Adventitious rooting (AR) is a crucial physiological process for propagating many horticultural plant species [11]. The formation of AR is a form of post-embryonic organogenesis, as new root tissues develop in locations other than the primary root system. Most researchers describe three or four sequential stages in the AR process: dedifferentiation, induction, initiation, and expression (emergence and outgrowth) [12]. Every step in the AR process is subjected to dynamic and specific hormone modulation, which provides a signalling network within the plant [13,14].

Auxin is a primary activating signal for AR induction [15]. Indole-3-acetic acid (IAA) biosynthesis and transport are crucial for cell fate transition from procambial cells to AR founder [16]. Numerous studies in *Arabidopsis* and other species, including rice, apple, and tomato have shown that upregulation of genes related to auxin biosynthesis (*YUC1*, *YUC3*, *YUCCA6*, *YUC10*, *AAO*, *TDC*), polar transport (*AUX1*, *LAX*, *PIN1*, *PIN2*, *PILS*, *MdPAT*, *MdPIN8*), auxin response factors (*ARFs*) and signal transduction (*GH3*) contributed to the auxin peak and thus to the induction or initiation of AR formation [11,15,17]. In horticultural practice, auxin must be applied exogenously to induce AR formation. However, many plant species cultured in vitro, including rhubarb, exhibit recalcitrant behaviour in response to different exogenous auxin treatments [8,18,19].

It is known that the auxin effect on rooting is modulated by several factors, among which ethylene (ET) plays an important role. This gaseous plant growth regulator is described as being involved in the rooting process of various plant species by controlling auxin synthesis, transport, and action [20]. Ethylene acts in a concentration-dependent and phase-specific manner, either promoting or inhibiting AR formation [21]. For example, ET inhibited AR formation in *Prunus persica* and *Malus domestica* [22,23]. On the other hand, there are species such as petunia [24] and *Arabidopsis* [25], in which ET stimulates AR formation. In chestnut, the effect of ET depended on the ontogenetic state of the tissues [26].

The physiological mechanisms and hormone interactions that determine root formation in rhubarb in vitro are unknown. The present study aimed to assess the role of ethylene and its interaction with auxin in the formation of ARs in the valuable rhubarb Malinowy selection. To evaluate the effect of ET on root system development, we applied a precursor of ET biosynthesis—1 aminocyclopropane-1-carboxylic acid (ACC); an inhibitor of ET synthesis—aminoethoxyvinylglycine (AVG); and an inhibitor of ET action—silver nitrate (AgNO_3_). Changes in endogenous hormone levels and expression of genes involved in auxin, ethylene, and ABA biosynthesis, metabolism, and signalling were evaluated. Identifying and characterizing the factors that regulate AR formation in rhubarb are essential for understanding and potentially manipulating the rooting process under in vitro conditions.

## 2. Results

### 2.1. Adventitious Root Formation in Response to IBA and Ethylene

**Figure 1:**
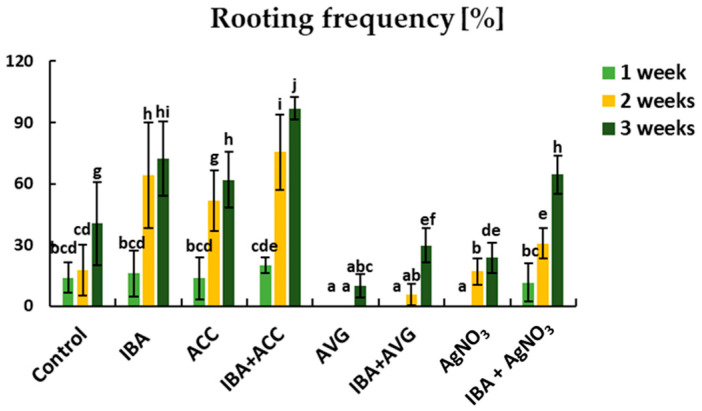
Effect of IBA and ethylene on rooting frequency of rhubarb ‘Raspberry’ selection after a 1-, 2-, and 3-week rooting period. Bars represent means ± SE; means indicated with the same letter within each rooting time do not differ significantly according to Duncan’s test (*p* ≤ 0.05) (*n* = 30).

**Figure 2:**
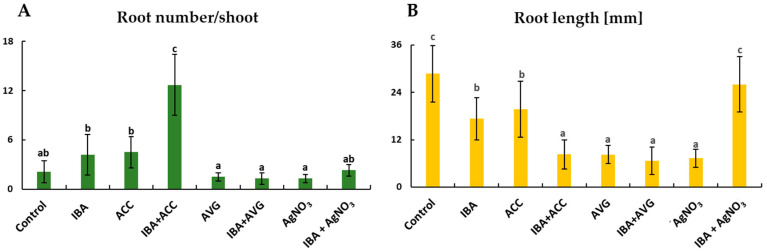
Effect of IBA and ethylene on root number (**A**) and length (**B**) of rhubarb ‘Raspberry’ selection after a 3-week rooting period. Bars represent means ± SE; means indicated with the same letter do not differ significantly according to Duncan’s test (*p* ≤ 0.05) (*n* = 30).

**Figure 3:**
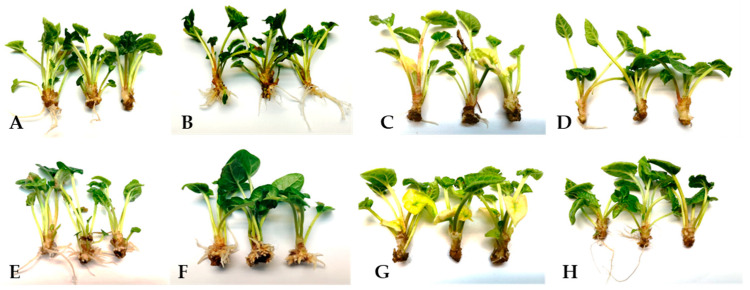
The rhubarb plantlets after a 3-week rooting period on media supplemented with different growth regulators: (**A**) Control, (**B**) ACC, (**C**) AVG, (**D**) AgNO_3_, (**E**) IBA, (**F**) IBA + ACC, (**G**) IBA + AVG, (**H**) IBA + AgNO_3_.

To determine the effect of ethylene on rhubarb AR formation, the microshoots were treated with a precursor of ethylene biosynthesis—1 aminocyclopropane-1-carboxylic acid (ACC), an inhibitor of ethylene synthesis—aminoethoxyvinylglycine (AVG), or an inhibitor of ethylene action—AgNO_3_. Morphological observation revealed the emergence of AR on the shoots within 1 to 3 weeks (Figure 1). On a hormone-free medium (control), a maximum of 40.5% of rooted shoots were obtained after 3 weeks (Figure 1). Both auxin and ACC promoted AR formation, increasing the rooting frequency to 72.2% and 61.9%, respectively. However, the highest rooting percentage (96.9%) and root number per shoot (12.7) were obtained on medium supplemented with both IBA and ACC (Figure 1 and Figure 2). The addition of ACC to the auxin-containing medium resulted in a 3-fold increase in the number of roots and a 2-fold decrease in root length. The inhibitors of ethylene synthesis and perception reduced the rooting efficiency, but to varying degrees. The lowest rooting percentage was observed when shoots were growing in the presence of AVG alone (12%) or in combination with auxin (29.8%) (Figure 1). Moreover, the rhubarb shoots treated with AVG were characterized by loss of chlorophyll content (Figure 3). After adding AgNO_3_ to the auxin medium, the rooting frequency was 64.4%. This was only 8% less than under the influence of auxin alone. On the other hand, the presence of silver ions in the IBA medium significantly reduced the number of roots per shoot and stimulated the root length (Figure 1 and Figure 2). The application of AgNO_3_ singly resulted in a decrease in rooting frequency by 16.7% compared to the control (without auxin).

### 2.2. Changes in the Phytohormone Contents in Rhubarb Shoots

To investigate the mechanism by which ethylene promotes root response in rhubarb microshoots, we examined the effects of auxin and the regulators of ethylene synthesis and action on endogenous hormone levels. The analysis revealed that after 1 week of growth on hormone-free medium, rhubarb shoots exhibited high levels of m-Topoline (Table 1). After one week of rooting, mT content was significantly reduced by ACC treatments. When the stimulator of ethylene synthesis was added alone, the amount of mT in the shoot bases decreased by half compared to the control. However, adding ACC to the auxin-containing medium resulted in a 176% reduction in the mT level. Among endogenous cytokinins, the most abundant was izopentyladenine (IPA), followed by *cis*-zeatin riboside (cZR) and *trans*-zeatin (tZ). After one week of rooting free form accounted 47% of total endogenous CKs, and 59.9% after two weeks of rooting. The relative abundance of different metabolites analysed varied depending on the exogenous hormone treatments and rooting time. On the control medium, high levels of CKs persisted throughout the rooting period. However, the rhubarb shoots grown on media containing IBA and ACC showed a 345% decrease in IPA level after two weeks of rooting (Table 1). Moreover, after ACC supplementation increased the transport form of CKs in rhubarb shoot bases. The lowest cytokinin content was observed in the presence of IBA and AgNO_3_ after 1 week of rooting. On the other hand, silver ions caused a significant increase in the cytokinin content (tZ, IPA) in the shoot bases over the next week, whereas in IBA + ACC treatment, its level decreased. The increase in tZ and IPA levels after two weeks of rooting was also stimulated by the addition of AVG to the IBA-containing medium (Table 1).

**Figure 4:**
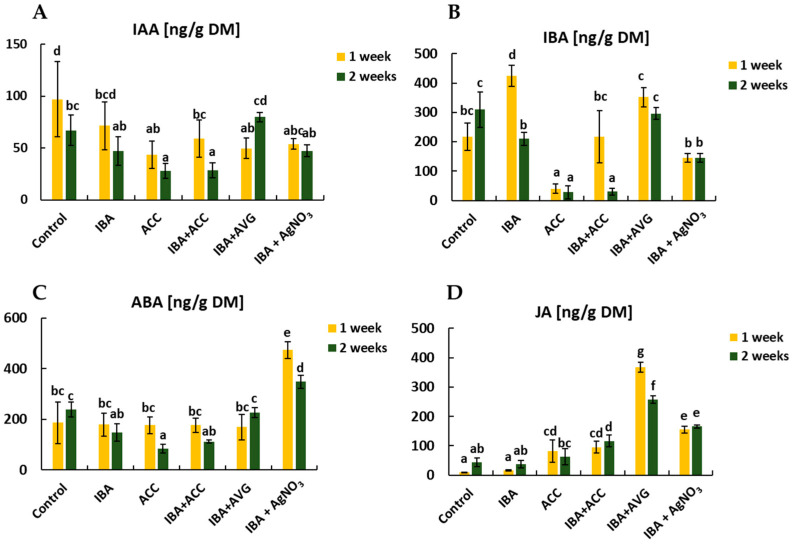
Changes in the content of endogenous hormones (ng·g^−1^ dry mass) in the rhubarb ‘Raspberry’ selection in weeks one and two of the rooting period (**A**—IAA content; **B**—IBA content; **C**—ABA content; **D**—JA content). Bars represent means ± SE; values marked with the same letter within each endogenous hormone do not differ significantly according to Duncan’s test (*p* ≤ 0.05) (*n* = 3).

The ABA content was the highest (474 ng·g^−1^ DM) after a one-week rooting period on a medium containing IBA and AgNO_3_. Over the next week, the concentration of ABA decreased (from 350 g·g^−1^ DM) but remained at a high level, 134.9% and 46.2% higher than in shoots grown on IBA and hormone-free medium, respectively. In contrast, ACC reduced the ABA levels. The shoots treated with ACC contained 182% and 112% less ABA than those grown on hormone-free and IBA medium, respectively. The shoots cultured in the presence of IBA or ACC alone showed a decreasing trend in ABA level as the rooting process progressed. The opposite trend was observed in shoots grown on hormone-free medium or supplemented with IBA and AVG (Figure 4).

As shown in Figure 4, blocking ethylene synthesis significantly enhanced JA production. After adding AVG to IBA-containing medium, the JA levels were 23- and 7-fold higher than those in shoots grown on IBA alone in weeks one and two of rooting induction, respectively. This was consistent with the low rooting rate.

### 2.3. Changes in the Expression of Genes Related to Ethylene, Auxin, and ABA Metabolism

**Figure 5:**
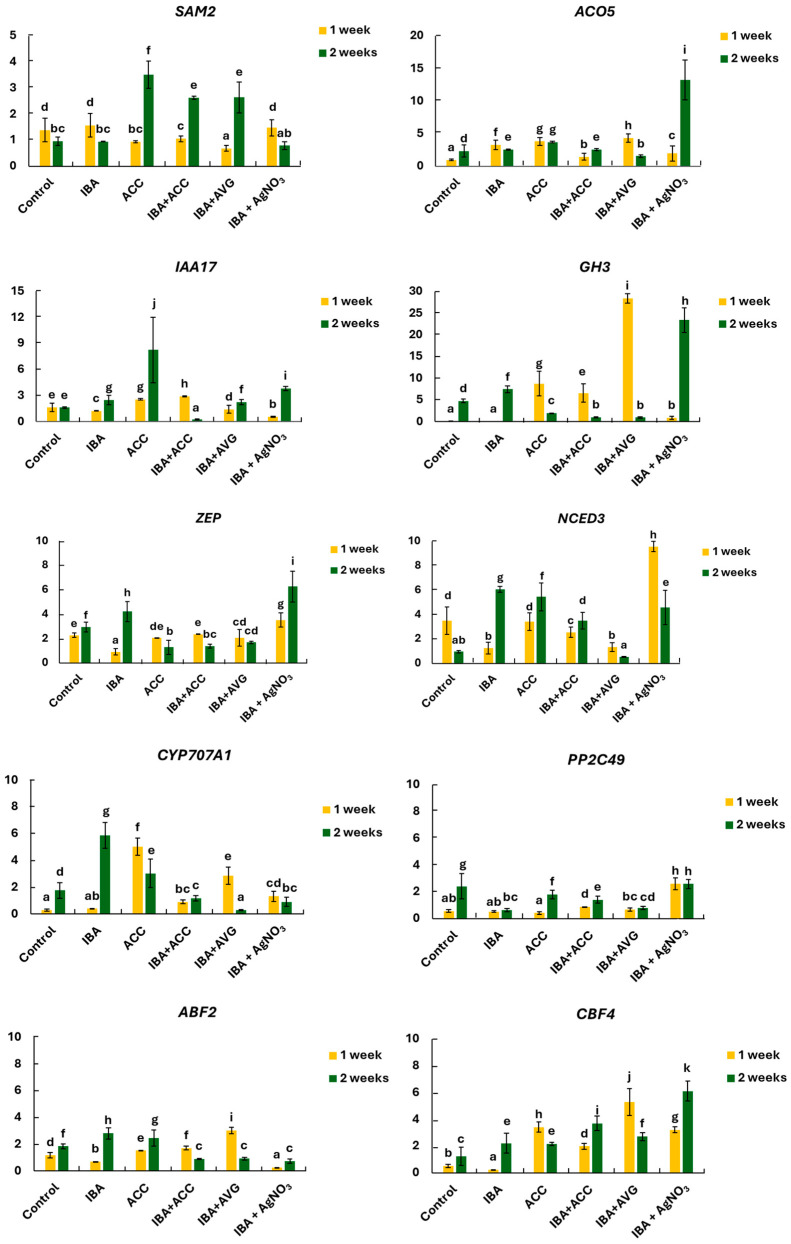
The relative expression of the genes involved in ethylene, auxin, and abscisic acid metabolism in rhubarb ‘Raspberry’. According to Duncan’s test (*p* = 0.05), means marked with the same letter do not differ significantly (*p* = 0.05). Error bars represent standard deviation.

As shown in Figure 5, the relative expression levels of two genes encoding enzymes responsible for ethylene synthesis, such as S-adenosylmethionine synthase 2 (*SAM2*) and ACC-oxidase 5 (*ACO5*), vary depending on the treatment. The expression of *SAM2* was the highest after a two-week rooting period in the presence of ACC (Figure 5), reaching a level 3.6 times higher than that in the control. *SAM2* expression was also high in the presence of IBA added together with ACC or AVG. The lowest *SAM2* activity was observed in shoots grown in the presence of IBA and AgNO_3_. In turn, the expression of *ACO5* increased after one week of rooting in shoots growing on different media, including IBA, ACC, and IBA + AVG. However, the highest increase in gene expression, 5.6-fold higher than the control, was observed after two weeks of growth in the presence of IBA and AgNO_3_.

The genes involved in auxin signalling, such as *IAA17*, which functions as a transcriptional repressor of early auxin response, and *GH3*, which encodes auxin amide synthetase responsible for regulating auxin homeostasis, were upregulated following ACC treatment. After one week of rooting, the highest increase in *GH3* gene expression (283.8-fold compared to the control) was observed in shoots treated with IBA and AVG. However, after two weeks of rooting, *GH3* expression was strongly induced in shoots treated with IBA and AgNO_3_ (Figure 5).

The genes involved in ABA synthesis, encoding zeaxanthin epoxidase (*ZEP*) and 9-cisepoxycarotenoid dioxygenase 3 (*NCED3*), were up-regulated by different treatments, including IBA and ACC. However, the highest relative expression level of both genes was observed in shoots growing on medium supplemented with IBA and AgNO_3_ (Figure 5). The gene encoding ABA 8′-hydroxylase 1 (*CYP707A1*), responsible for ABA catabolism, was strongly overexpressed one week after ACC and IBA + AVG treatment, with levels approximately 18- and 10-fold higher than in the control, respectively. After two weeks of rooting, *CYP707A1* expression was the highest (3.3 times higher than in the control) in shoots growing on the medium containing IBA only. Among the genes related to ABA signalling, the *PP2C49* exhibited strong overexpression after one week of growing in the presence of IBA and AgNO_3_ (Figure 5). The *ABF2* and *CBF4* genes, also involved in the ABA signalling, showed distinctly different expression patterns. The highest increase in *ABF2* expression (2.6-fold higher than the control) was observed after one week of rooting on a medium containing IBA and AVG. On the other hand, *CBF4* was strongly upregulated in response to different ethylene treatments. After one week of rooting, the highest *CBF4* expression (10.4-fold higher than the control) was observed in shoots treated with IBA and AVG. However, during the second week, the presence of silver ions in the auxin medium significantly enhanced *CBF4* expression (Figure 5).

## 3. Discussion

Adventitious rooting is a crucial stage in the micropropagation of many woody and perennial plant species. Our previous study showed that the auxin signal was insufficient to achieve effective in vitro rooting of rhubarb Raspberry type in the first rooting cycle [8]. Here, for the first time, it has been demonstrated that ethylene (ET) plays an essential role in AR formation in rhubarb in vitro. Adding ACC to the IBA-containing medium significantly increased the rooting rate and the number of roots during the first rooting cycle. Interestingly, the ethylene precursor applied alone also slightly improved rooting compared to the control. However, blocking ethylene synthesis significantly inhibited the AR formation. Additionally, we observed that ET content and signalling modulation are directly involved in ET synthesis. Particularly, ACC treatment showed a significant increase in the expression of the *SAM2* gene, which codes an 1-aminocyclopropane-1-carboxylate synthase that catalyzes the conversion of S-adenosyl-1-methionione (SAM) to ACC. On the other hand, a significant increase in *ACO5* expression (more than 4-fold higher than in the control) was observed after adding the silver ion to the IBA-containing medium. This may suggest a negative feedback mechanism—when ethylene perception is blocked, the plant, sensing an ethylene deficit, upregulates the expression of ethylene biosynthesis genes (particularly *ACS*) [27,28]. AgNO_3_ may also act as a stress factor, inducing the transcription of ethylene biosynthesis-related genes, including *ACO* genes [29]. While ethylene is known to interact with auxin during AR formation in different plant species, the mechanism of its action remains unclear.

In this study, we found that the ACC-induced reductions in cytokinin levels were coincident with enhanced rooting response. The inhibitory effect of high cytokinin levels during early rooting stages has also been observed in other plant species, including apple rootstocks [30], tomato [31], and *Cyclocarya paliurus* [14]. Studies on *Dianthus caryophyllus* revealed that genotypes with higher trans-zeatin levels tend to have lower rooting ability [32]. It has been demonstrated that cytokinins slow down the differentiation of primordia and AR formation by restricting the expression and transport of auxin-related genes (e.g., *MdYUCCA1*, *MdYUCCA10*, *MdAUX1*, *MdPIN1*, *MdPIN2*, and *MdPIN3*), thereby suppressing the auxin signalling pathway [30,33].

In plant tissue culture, CKs in the explant come from both internal production and the external supply in the medium. The process of taking in and breaking down both types of CKs determines the total CKs available in the explant, which in turn influences the plant’s growth and development in vitro [34,35]. Additionally, the metabolism of endogenous CKs can be significantly affected by exogenous CKs such as BA, mT, or TDZ present in the culture medium [36]. It is known that exogenous cytokinins used during shoot multiplication may have a negative influence on subsequent rooting and acclimatization [37,38]. In our micropropagation method, we apply mT, as we found it was most effective in rhubarb shoot formation. Numerous studies reported that shoots multiplied in the presence of mT exhibited a higher rooting ability than those grown in the presence of BAP and TDZ. In *Spatyphyllum*, Werbrouck et al. [39] demonstrated that mT is much faster degraded than BAP and does not accumulate in the shoot base. The high level of mT observed in rhubarb shoot bases after one week of rooting on the control medium may suggest its slow degradation. It seems interesting to investigate the subsequent effects of other cytokinins in the future. In this study, when ACC was added to the IBA-containing medium, the mT content in the rhubarb shoot bases decreased by 176%.

This study also provides the first information on the endogenous CKs composition of rhubarb shoots in vitro. It was shown that in rhubarb shoot bases during rooting, IPA dominated, followed by cZR and tZ. Similarly to other plant species, including *Morus alba* and *Dianthus caryophyllus* [32,40], we observed that low concentrations of CKs were conducive to AR formation. A significant decrease (by 345%) in IPA level was obtained after adding ACC to the IBA medium. It was somewhat surprising to find the lowest cytokinin content in rhubarb treated with IBA and AgNO_3_. A recent study showed that AgNO_3_, as in the case of reactions to heavy metals, can act as a stress factor and thus disrupt hormonal balance. The plant may shift its metabolism towards defence pathways (JA/SA or ABA) at the expense of cytokinin biosynthesis [29,41,42]. Similarly, we observed a simultaneous increase in ABA level and upregulation of genes related to ABA biosynthesis and signalling (*PP2C49* and *CBF4*), and downregulation of genes related to ABA catabolism (*CYP707A1*).

In addition, exogenous ethylene application in rhubarb significantly decreased endogenous auxin levels (IAA and IBA) in the shoot bases during the later rooting stage, thereby increasing rooting frequency and root number. However, blocking ethylene synthesis produced the opposite effect. It is known that the auxin response depends on its concentration, and optimal levels differ at each stage of AR development [21]. Numerous studies have shown that high auxin concentrations are necessary for AR induction [11,31]. Nevertheless, in later stages, elevated auxin levels can inhibit the differentiation and outgrowth of root primordia [43]. Unfortunately, this study does not provide data on auxin levels in the shoot bases of rhubarb immediately after shoot excision (the induction stage of AR formation). Based on observations of rhubarb explants, we assume that the endogenous hormone levels and gene expression after 1 and 2 weeks correspond to the initiation and expression stages of rhubarb AR formation, respectively. It has been reported that auxin homeostasis is maintained through a complex network involving biosynthesis, polar transport, metabolism, and conjugation [15,16]. Indole-3-acetic acid (IAA) is the plant’s primary active form of auxin. In addition to de novo biosynthesis, IAA can be released from its conjugates with sugars and amino acids or through the conversion of indole-3-butyric acid (IBA) [44]. This study showed that IBA was the dominant auxin in rhubarb shoot bases during the later rooting stage. Its high concentration was also observed in the control, indicating that it was not due to exogenous stimuli. Endogenous IBA has been detected in many plant species, but its concentration is often lower than that of IAA (reviewed in [45]). An efficient conversion of IBA to IAA is critical for AR formation in various species, including *Prunus subhirtella* ‘Autumnalis’ and *Cyclocarya paliurus* [14,46].

This study revealed a positive relationship between ET-induced reductions in IAA and IBA levels in rhubarb shoot bases, observed at both the initiation and expression stages, and the enhanced rooting response. In *Arabidopsis*, ET was shown to promote the formation and growth of ARs by downregulating genes related to IAA biosynthesis (e.g., *WEI2*, *WEI7*, and *YUC6*), while also enhancing the conversion of IBA into active IAA [25]. We suppose that the reduced levels of IBA in rhubarb shoot bases following ACC treatment may also result from the conversion of IBA to IAA. This may suggest, in the presence of ET, enhanced expression of the auxin-inducible *Gretchen Hagen 3* (*GH3*) gene, *GH3.1*. Members of the GH3 protein family have IAA-amido synthetase activity, which catalyzes the conjugation of active IAA to amino acids such as aspartic acid and glutamic acid [47]. Many studies have demonstrated that this mechanism plays a crucial role in the negative feedback regulation of IAA concentration, with excess IAA upregulating *GH3* expression [17]. Similarly to the results presented for *Arabidopsis* [48,49], we observed a clear relationship between *GH3* expression levels and activation of AR initiation in rhubarb shoots. To date, the *GH3.1* gene has not been reported to be involved in the conjugation of IBA to amino acids. However, other isoforms, such as *GH3.15*, have been shown to be specific to IBA [45,50].

In addition, we showed that blocking ethylene synthesis significantly increased JA production and *GH3.1* expression in the rhubarb shoot base during the root initiation stage. In this case, enhanced *GH3.1* activity was associated with a poor rooting response. JA is a typical stress-related hormone classified as a rooting inhibitor that works downstream of the IAA pathway [51]. Some authors reported that early wound-induced JA accumulation in cuttings can stimulate AR induction via enhanced IAA accumulation [21,52]. In our study, it remains unclear whether the high JA level under AVG treatment was due to blocked ethylene synthesis or was a stress response to AVG, as suggested by the observed leaf senescence. As mentioned, AVG-stimulated high JA production coincided with high *GH3.1* activity after one week of rhubarb rooting. In *Arabidopsis*, it has been shown that certain auxin-responsive *GH3* genes also regulate JA homeostasis. Gutierrez et al. [51] found that *GH3.3*, *GH3.5*, and *GH3.6* genes contribute to JA conjugation during auxin-stimulated AR formation. On the other hand, *GH3.11*, also known as JASMONIC ACID RESISTANT1 (*JAR1*), inhibits AR formation in *Arabidopsis* [53]. Recently, *AtGH3.10* was reported to activate JA biosynthesis and functions partially redundantly with *AtJAR1* in the wound stress response [54]. Our results suggest that high *GH3.1* activity may have directly affected JA biosynthesis or indirectly through auxin levels. Interestingly, only the AVG treatment caused an increase in auxin levels during the next step of rhubarb rooting, which was consistent with reduced *GH3.1* activity and decreased, but still high, JA levels. At this research stage, it is difficult to determine whether ethylene mediates the cross-talk between auxin and JA in regulating hormone homeostasis and promoting AR formation in rhubarb shoots in vitro.

In turn, the presence of silver ions on the IBA medium caused a significant increase in ABA levels in the rhubarb shoot bases during the late rooting stages, thereby inhibiting AR formation. ABA is known as the key regulator of dormancy. It has been shown that high endogenous ABA levels result in low rooting efficiency in vitro and poor survival and early growth ex vitro of some woody and herbaceous perennials, including walnut, magnolia, peony, and rhubarb [19,55,56]. Bouza et al. [57] observed that the accumulation of ABA in shoots was induced in response to exogenous IBA application during the rooting of peony plantlets. In this study, IBA supply induced the expression of ABA biosynthesis genes (*ZEP* and *NCED3*) and enhanced the ABA catabolism gene (*CYP707A1*). The addition of ACC to the IBA-containing medium suppressed the expression of both ABA biosynthesis and catabolism genes. The high ABA contents in the rhubarb shoot bases in the presence of IBA and AgNO_3_ were consistent with upregulation of *PP2C49* and *CBF4* (genes related to ABA biosynthesis and signalling) and downregulation of *CYP707A1*, related to ABA catabolism. Many studies showed that ABA and ethylene interact antagonistically at multiple levels, affecting each other’s synthesis and signal transduction pathways [58]. For example, in rice, ABA negatively controls AR initiation and development by blocking ethylene signalling [59,60]. It is widely recognized that the nature of the ET-ABA interaction is strongly dependent on the endogenous ABA and ethylene levels, varying by tissue type, developmental stage, plant species, and environmental conditions [58]. Our results indicate that certain ethylene levels are essential for maintaining low ABA levels and their response during auxin-controlled AR formation in rhubarb in vitro.

## 4. Materials and Methods

### 4.1. Plant Material

The garden rhubarb ‘Malinowy’ cultivar, known for its high levels of anthocyanin, raponticin, and resveratrol in the leaf petioles, was used in the study. In vitro shoot cultures were established and continuously propagated every 3–4 weeks on Murashige and Skoog (MS) medium [61], modified with a quarter dose of nitrogen salts, supplemented with 3 mg·L^−1^ hydroxybenzylaminopurine (meta-Topolin, mT), and 1.0 mg·L^−1^ gibberellic acid (GA_3_) [8]. In the final subculture before rooting, shoots were grown on a medium containing 1.5 mg·L^−1^ meta-topolin.

### 4.2. Effect of Auxins and Ethylene on In Vitro Rooting

For in vitro rooting, well-developed shoots (approx. 4 cm) were cultured on a modified MS medium containing 50% nitrogen salts. All culture media included 100 mg·L^−1^ myo-inositol, vitamins (nicotinic acid, pyridoxine, thiamine (1.0 mg·L^−1^ each), 2 mg·L^−1^ glycine, 30 mg·L^−1^ sucrose, and 6.5 g·L^−1^ agar (Plant Propagation Lab-Agar, BioMaxima, Lublin, Poland), pH 5.8. The shoots were maintained at 20 ± 2 °C under a standard 16/8 h photoperiod provided by cool-white fluorescent lamps at 40–50 µmol m^−2^ s^−1^.

To evaluate the role of ethylene in adventitious root formation in rhubarb in vitro, we initially tested whether the presence of ethylene in the medium would affect the rooting rate. Single rhubarb shoots were cultured on media without growth regulators (control), supplemented with auxin (IBA), a precursor of ethylene biosynthesis (1-aminocyclopropane-1-carboxylic acid, ACC, 2 mg·L^−1^), an inhibitor of ethylene synthesis (aminoethoxyvinylglycine, AVG, 1 mg·L^−1^), or an inhibitor of ethylene action (AgNO_3_, 1 mg·L^−1^). Auxin and ethylene regulators were used alone or in combination (IBA + ACC, IBA + AVG, IBA + AgNO_3_).

The rooting frequency was determined after 1, 2, and 3 weeks of culture. The number and length of roots were assessed after 3 weeks. Additionally, after 1 and 2 weeks of rooting from some treatments (Control, ACC, IBA, IBA + ACC, IBA + AVG, IBA + AgNO_3_), 12 randomly selected shoots were collected to evaluate the content of endogenous hormones (IAA, IBA, ABA, JA) and gene expression. The basal parts (0.5 cm) of the microshoots were cut off and pooled in 3 technical samples.

### 4.3. Quantification of Aux, ABA, and JA

Immediately after collection, the rhubarb shoot bases were frozen in liquid nitrogen and then lyophilized and homogenized. Each sample used 50 mg of ground plant material. We extracted phytohormones using a 1 mL mixture of methanol, water, and formic acid (15:4:1; *v*/*v*/*v*) as described by Dobrev and Kaminek [62], with modifications by Stefancic et al. [63]. We added an internal isotopic standard mixture of deuterated IAA, JA, ABA, and ^15^N labelled t-zeatin (^15^N-t-Z) to each sample. After centrifuging the extract, we collected the supernatant and repeated the extraction. We combined the supernatants, dried them, and reconstituted them in 1 mL of 1 M formic acid. This extract was then fractionated using SPE columns (Oasis MCX, 1 cc/30 mg, Waters Corporation, Milford, MA, USA).

Acidic fractions were eluted from the SPE column with 1 mL of methanol, evaporated to dryness, and then reconstituted in 50 μL of methanol. Samples prepared this way were analyzed on an HPLC column, Supelco Ascentis RP-Amide (7.5 cm × 4.6 mm, 2.7 μm). The mobile phases used were 0.1% formic acid in water (solvent A) and a 1:1 mixture of acetonitrile and methanol. Gradient elution was applied at a flow rate of 0.5 mL/min. The HPLC apparatus was an Agilent Technologies 1260 (Santa Clara, CA, USA) equipped with an Agilent Technologies 6410 Triple Quad LC/MS with ESI (Electrospray Interface). For the most analyzed compounds, the two most abundant secondary ions (MRM—multiple reaction monitoring modes) were monitored. One ion was used for quantification (the quantifier ion), while the second was used for additional identity confirmation (the qualifier ion). The monitored ions were: indole-3-acetic acid (IAA)—*m*/*z* 176.1 primary, 130.3, 77.2 secondary; indolebutyric acid (IBA)—*m*/*z* 204.1 primary, 186.4, 130.3 secondary; deuterated IAA (D-IAA, used as an internal standard)—*m*/*z* 181.1 primary, 134.7 secondary; abscisic acid (ABA)—*m*/*z* 265.2 primary, *m*/*z* 229.1, 247.1 secondary; deuterated ABA (D-ABA, used as an internal standard)—*m*/*z* 271.2 primary, *m*/*z* 167.1 secondary; jasmonic acid (JA) *m*/*z* 211.1 primary, 133.1, 151.1 secondary; and deuterated JA (D-JA, used as an internal standard) *m*/*z* 216.2 primary, 135.2 secondary. Calibration curves were prepared for the analyzed compounds, spanning 10 points.

### 4.4. Quantification of Cytokinins

Cytokinins, such as t-Z, c-Z, t-ZR, c-ZR, mT, oT, and iPA, were separated from the samples. Cytokinins were flushed out of the SPE column after collecting IAA, ABA, and JA; they were eluted with 0.35 M ammonia in 60% methanol [64] (the cleaning step with a water solution was omitted). The collected fraction was evaporated to dryness, reconstituted in 50 μL methanol, and analyzed using the same chromatographic system and HPLC column as described above. The solvent system consisted of water with 0.001% acetic acid (solvent A) and acetonitrile with 0.001% acetic acid (solvent B) at a flow rate of 1.5 mL·min^−1^, gradient profile was 2.5% B to 1 min, 10% B at 3 min, 25% B at 6 min, 75% B at 8 min and 2.5% B at 8.5 min. As for the above-described compounds, the two most abundant secondary ions (in multiple reaction monitoring modes, MRM) were monitored. One ion was used for quantification (the quantifier ion), while the second was used for additional identity confirmation (the qualifier ion). The monitored ions were: trans-zeatin (t-Z) and cis-zeatin (c-Z) *m*/*z* 220.1 primary, 136.1, 202.1 secondary; trans-zeatin ryboside (t-ZR) and cis-zeatin ryboside (c-ZR) *m*/*z* 352.2 primary, 136.1, 220.1 secondary; meta-topolin (mT) *m*/*z* 242.1 primary, 77.1, 107.0 secondary; orto-topolin (oT) *m*/*z* 242.1 primary, 107.0, 136.1 secondary; isopentenyladenine (iPA) *m*/*z* 204.1 primary, 136.1, 148.1 secondary; and ^15^N labelled t-zeatin (^15^N-t-Z, used as an internal standard for all cytokinines) *m*/*z* 224.1 primary, 140.1 secondary. Calibration curves were prepared for the analyzed compounds, spanning 10 points.

### 4.5. Molecular Analysis

Molecular studies involved analyzing the expression of genes associated with ABA, ethylene, and auxin metabolism. RNA extraction for this analysis was performed following the method described by Chang et al. [65]. DNA traces were removed from RNA samples by digestion with RQ RNase-Free DNase (Promega, Madison, WI, USA). Then, RNA samples were purified using the RNeasy Mini Kit (Qiagen, Hilden, Germany) according to the protocol for RNA clean-up. The concentration and purity of the total RNA were examined in duplicate using an Epoch spectrophotometer (BioTek, Highland Park, VT, USA). From each sample, one µg of RNA was reverse-transcribed using M-MLV reverse transcriptase (Promega, Madison, WI, USA) and oligo(dT)15 primer (Promega, Madison, WI, USA). The cDNA samples were used for gene expression analysis using the quantitative real-time PCR (qRT-PCR) technique with specific primers. Primers were designed based on gene sequences available in the literature [66,67,68,69]. For each gene, three primer pairs were tested, and PCR conditions were optimized. PCR reactions were carried out using DreamTaq DNA polymerase (Thermo Fisher Scientific, Waltham, MA, USA) with a pooled cDNA sample prepared from all analyzed materials as template. PCR products were separated by agarose gel electrophoresis, sequenced, and subjected to homology analysis to verify amplification specificity. In addition, melting curve analysis was performed (in the range of 72–95 °C) to confirm primer specificity. Relative expression was based on the glyceraldehyde-3-phosphate dehydrogenase (*GAPDH*) gene, which served as a reference gene [66]. Detailed information on the primers selected for the final analyses is presented in Table 2. Quantitative RT-PCR was carried out in a Rotor-Gene 6000 machine (Corbett Research, Bath, UK) using the KAPA SYBR Fast qPCR Master Mix (Kapa Biosystems, Amsterdam, The Netherlands), according to the manufacturer’s instructions, with a 1/10 dilution of cDNA for each tested sample. The annealing temperature for all primers was 58–60 °C, depending on the primers. Four ten-fold dilutions of cDNA were run with the analyzed samples to calculate the standard curve (correlation coefficient > 0.99) and determine PCR efficiency. The relative quantification of the mRNA levels of the tested genes was derived from the standard curve, normalized to the reference gene, and the control sample. Fold change was calculated using the standard 2^−ΔΔCt^ method.

### 4.6. Statistical Analysis

For in vitro experiments, 30 shoots (5 shoots × 6 glass jars) were used in each treatment. The experiments were carried out twice. The final data were the means of two replicated experiments. The data were subjected to a one-factor and two-factor analysis of variance using STATISTICA software (v. 13.3). Duncan’s test was performed to evaluate the significance of differences at *p* ≤ 0.05.

All the gene expression data were analyzed using Rotor-Gene 6000 Series Software 1.7 (Corbett Research, Bath, UK). Quantitative RT-PCR data represented the average of at least two independent biological replicates, each performed with three technical repetitions. Standard deviation indicates the variation between biological replicates. Microsoft Office 365 (Redmond, WA, USA) was used for creating the figures.

## 5. Conclusions

Our study revealed that the in vitro rooting of rhubarb shoots is a highly complex process controlled by multiple hormones. For the first time, we demonstrated that auxin-controlled AR formation in rhubarb can be modified by ET level, which changed the hormone balance. Adding ACC to the IBA-containing medium significantly increased the rooting rate and the number of roots during the first rooting cycle. It was found that rhubarb shoots taken to rooting were characterized by a high content of mT, which was overaccumulated during cyclic multiplication on the mT-medium. The positive effect of ethylene on rhubarb rooting was closely related to its capacity to decrease CK contents at the shoot bases during the initiation stage of AR formation. In addition, ET was shown to decrease auxin levels, which was consistent with the enhanced expression of the auxin-inducible *GH3.1* gene, indicating its indirect role in converting auxin to an inactive form. After adding AVG or AgNO_3_ to IBA medium, we observed a significant increase in the production of JA and ABA, respectively, as well as the upregulation of several genes involved in ABA biosynthesis and signalling. These results suggest that the regulation of AR formation in garden rhubarb in vitro by IBA and ethylene is also mediated through interactions with ABA and JA. Identifying and characterizing the factors that regulate AR formation and development is valuable and essential for potentially manipulating AR formation in different rhubarb varieties. However, understanding the whole mechanism of AR induction and development requires further study.

## Figures and Tables

**Table 1:** Changes in cytokinin content (ng·g^−1^ dry mass) in the rhubarb ‘Raspberry’ selection after 1 week (A) and 2 weeks (B) of rooting. Valuess are expressed asmeans ± standard error; values marked with the same letter within individual cytokinin do not differ significantly according to Duncan’s test (*p* ≤ 0.05) (*n* = 3).

Cytokinin (ng/g DM)	Time	Treatments					
		Control	IBA	ACC	IBA + ACC	IBA + AVG	IBA + AgNO_3_
free form *							
*trans*-zeatin (tZ)	A	1.4 ± 0.5 cd	0.7 ± 0.2 ab	2.4 ± 0.5 e	0.6 ± 0.2 a	0.4 ± 0.03 a	0.5 ± 0.1 a
	B	0.7 ± 0.1 ab	1.3 ± 0.2 bc	2.0 ± 0.4 de	0.5 ± 0.1 a	2.0 ± 0.2 de	5.0 ± 0.9 f
izopentyladenine (IPA)	A	3.7 ± 0.8 bc	2.4 ± 1.1 ab	4.1 ± 0.5 c	3.7 ± 0.7 bc	1.9 ± 0.5 a	2.1 ± 0.4 a
	B	7.8 ± 2.1 de	3.8 ± 0.8 bc	2.4 ± 0.5 ab	1.1 ± 0.3 a	8.9 ± 0.4 e	6.8 ± 0.9 d
dihydroxyzeatin (DZ)	A	0.2 ± 0.03 bc	0.1 ± 0.03 a	0.1 ± 0.03 a	0.04 ± 0.03 a	0.05 ± 0.00 a	0.1 ± 0.02 a
	B	0.1 ± 0.02 a	0.2 ± 0.1 b	0.3 ± 0.07 c	0.1 ± 0.01 a	0.2 ± 0.05 bc	0.1 ± 0.00 ab
*cis*-zeatin (cZ)	A	1.1 ± 0.4 ab	1.2 ± 0.2 ab	1.2 ± 0.3 ab	0.7 ± 0.1 a	1.4 ± 0.6 a–c	1.4 ± 0.3 a–c
	B	1.3 ± 0.4 ab	1.6 ± 0.4 ab	1.0 ± 0.3 ab	1.0 ± 0.1 ab	1.0 ± 0.2 ab	1.9 ± 0.2 c
orto-Topolin (oT)	A	0.4 ± 0.1 b	1.0 ± 0.5 c	0.3 ± 0.06 ab	0.2 ± 0.06 ab	0.1 ± 0.02 ab	0.3 ± 0.09 ab
	B	0.4 ± 0.1 b	0.2 ± 0.01 ab	0.1 ± 0.03 ab	0.02 ± 0.0 a	0.02 ± 0.0 a	0.1 ± 0.02 ab
conjugated form *							
*trans*-zeatin riboside (tZR)	A	0.8 ± 0.2 a–c	0.2 ± 0.03 a	1.2 ± 0.4 cd	0.3 ± 0.1 a	0.4 ± 0.2 ab	0.2 ± 0.05 a
	B	0.3 ± 0.2 ab	1.6 ± 0.4 d	3.9 ± 1.0 e	1.0 ± 0.3 bd	0.4 ± 0.1 ab	0.3 ± 0.04 ab
dihydroxyzeatin riboside (DZR)	A	0.3 ± 0.1 a–c	0.1 ± 0.05 a	0.3 ±0.1 a–c	0.2 ± 0.1 ac	0.1 ± 0.0 a	0.1 ± 0.00 a
	B	0.2 ± 0.04 ac	0.4 ± 0.1 c	1.0 ± 0.3 d	0.4 ± 0.1 c	0.1 ± 0.01 ab	0.2 ± 0.03 a–c
*cis*-zeatin riboside (cZR)	A	5.5 ± 0.8 bc	6.0 ± 1.6 bc	3.2 ± 0.5 a	6.2 ± 1.9 c	3.9 ± 0.2 ab	3.9 ± 0.3 ab
	B	4.6 ± 1.4 a–c	4.4 ± 0.9 a–c	3.0 ± 0.3 a	5.8 ± 2.0 bc	3.1 ± 0.3 a	2.4 ± 0.5 a
**Total** ** endogenous cytokinin **	A	13.4 ± 1.9 cd	11.7 ± 2.0 bc	12.6 ± 1.5 bcd	11.7 ± 2.3 bc	8.3 ± 1.4 a	8.4 ± 3.0 a
	B	15.3 ± 2.8 cde	13.1 ± 1.6 bcd	13.7 ± 1.4 cde	9.8 ± 1.9 ab	15.7 ± 3.0 de	16.9 ± 5.7 e
meta-Topolin (mT)	A	438 ± 85.2 f	205 ± 17 de	240 ± 65 e	116 ± 19.9 ab	227 ± 29.7 e	81.9 ± 1.6 a
	B	129 ± 20 a–c	159 ± 20 b–d	112 ± 10 ab	91 ± 18.2 a	141 ± 19 a–d	187 ± 3 b–e
**Total**	A	451.2 ± 88 g	216.9 ± 21 ef	252.8 ± 68 f	128.0 ± 23 a–c	235.6 ± 31 f	90.4 ± 3.0 a
	B	143.9 ± 26 a–d	171.6 ± 22 c–e	126.0 ± 13 a–c	100.4 ± 21 ab	158 ± 21 b–e	204 ± 5.7 d–f

* cytokinins listed in order of their relative strength.

**Table 2:** Sequences of the primer pairs used for the real-time PCR analysis.

Gene	Sequence
*ACO5* (according to Iwamoto et al. 2010 [69])	5′-CCGAAGGAGCTTCTTGATCGG-3′
	5′-ATTTTGGCGCCTTGACGGCC-3′
*SAM2* (according to Mala et al. 2021 [66])	5′-CATGCCCCTTAGCCACGTT-3′
	5′-GGTCTTGCCATCAGGCCTTA-3′
*IAA17* (according to Mishra et al. 2009 [68])	5′-CAAATCCAGATCAAAACACAGACAA-3′
	5′-GGTGTTAATTGCTCTTTTTTTTCTTACG-3′
*GH3* (according to Mishra et al. 2009 [68])	5′-CCCACAGTGAAAAAAAACGAGTAA-3′
	5′-CTTGCTGGTGCTTTAGTTTTTCTTC-3′
*ZEP* (according to Mala et al. 2021 [66])	5′-GGCACAAGGGATCACGAACT-3′
	5′-CCTTGGAGGAGAATCGAATGG-3′
*NCED3* (according to Zhang et al. 2021 [67])	5′-TCGAAGCAGGGATGGTCAAC-3′
	5′-CCTGAGACTTTAGGCCACGG-3′
*CYP707A1* (according to Zhang et al. 2021 [67])	5′-CACTGAAGAGCAAGAGGCTATA-3′
	5′-TTCTTGGTATCTGCCCAACTC-3′
*PP2C49* (according to Mala et al. 2021 [66])	5′-GATCGACGACCTATCCATGCA-3′
	5′-GGTCCTCCATGGCCATCA-3′
*ABF2* (according to Zhang et al. 2021 [67])	5′-TCGTTGACTCTGCCTCGAAC-3′
	5′-CCTGAGCCACCTGAGACAAG-3′
*CBF4* (according to Zhang et al. 2021 [67])	5′-GATGATGAGGCGCTTTTGGG-3′
	5′-TCACCCACTCCGTCAAAGTC-3′
*GAPDH* (according to Mala et al. 2021 [66])	5′-CTCAATGACGGCCACACAGA-3′
	5′-ACCAGTGCTGCTGGGAATG-3′

## Data Availability

All data are included in this article.
